# Cerebral infarction related to nonbacterial thrombotic endocarditis in a middle-aged woman with uterine adenomyosis: A case report

**DOI:** 10.1097/MD.0000000000033871

**Published:** 2023-06-02

**Authors:** Jeong-Sook Seo

**Affiliations:** a Division of Cardiology, Department of Internal Medicine, Busan Paik Hospital, Inje University College of Medicine, Busan, South Korea.

**Keywords:** adenomyosis, case report, cerebral infarction, hysterectomy, nonbacterial thrombotic endocarditis, thromboembolism

## Abstract

Few isolated case reports and case series have reported arterial and venous thromboembolism related to adenomyosis; however, the underlying mechanism remains unclear.

**Patient concerns:**

A 47-year-old woman presented with dizziness, nausea, vomiting, and loss of consciousness after red blood cell transfusion. She was being treated for menorrhagia and severe anemia.

**Diagnoses:**

Magnetic resonance imaging showed multiple infarctions in right cerebellum and bilateral frontal, parietal, and occipital lobes. Echocardiography performed during the evaluation for the source of emboli revealed multiple echogenic masses on the tricuspid aortic valve. There was no evidence of infection, and the masses on the aortic valve were diagnosed as nonbacterial thrombotic endocarditis. The levels of autoimmune antibodies and tumor markers except for carbohydrate antigen 19-9 and cancer antigen 125 were within the normal range. Uterine ultrasound showed a large adenomyosis. The patient was diagnosed with multiple cerebral and cerebellar infarctions due to nonbacterial thrombotic endocarditis, and hormone therapy and anticoagulation with warfarin were initiated.

**Interventions:**

The patient did not develop recurrent infarction during anticoagulant therapy; however, menorrhagia worsened requiring total hysterectomy.

**Outcomes:**

The patient did not experience recurrent infarction despite the absence of anticoagulant therapy during the 3-year follow-up period.

**Lessons:**

The present case adds to the limited number of previously reported cases and supports that, albeit rare, adenomyosis can be associated with embolic infarction and suggests that nonbacterial thrombotic endocarditis might be the link between adenomyosis and embolic infarction.

## 1. Introduction

Adenomyosis, which is a benign disease most commonly diagnosed in middle-aged women, is characterized by abnormal endometrial growth that presents with symptoms including menorrhagia, dysmenorrhea, and chronic pelvic pain. Few isolated case reports and case series have reported arterial and venous thromboembolism related to adenomyosis; however, the underlying mechanism remains unclear. Here we report the case of a patient with adenomyosis who experienced embolic cerebral and cerebellar infarction associated with nonbacterial thrombotic endocarditis (NBTE) on the aortic valve.

## 2. Case presentation

A 47-year-old woman was transferred to our hospital with non-whirling dizziness, nausea, vomiting, and loss of consciousness after the transfusion of red blood cells. She was admitted to the previous hospital due to menstruation lasting more than a week and diagnosed with severe anemia based on a hemoglobin level of 3.4 g/dL. She had no risk factors for atherosclerotic vascular disease such as hypertension, diabetes mellitus, dyslipidemia, or smoking.

At arrival, her vital signs were as follows: blood pressure, 120/80 mm Hg; pulse rate, 66 beats/min; and body temperature, 36.8℃. Her body weight and height were 51 kg and 158 cm, respectively. The finger-to-nose test and the rapid alternating movement test with the left hand were intact; however, her right hand was inaccurate and slow in both tests.

**Figure 1.**
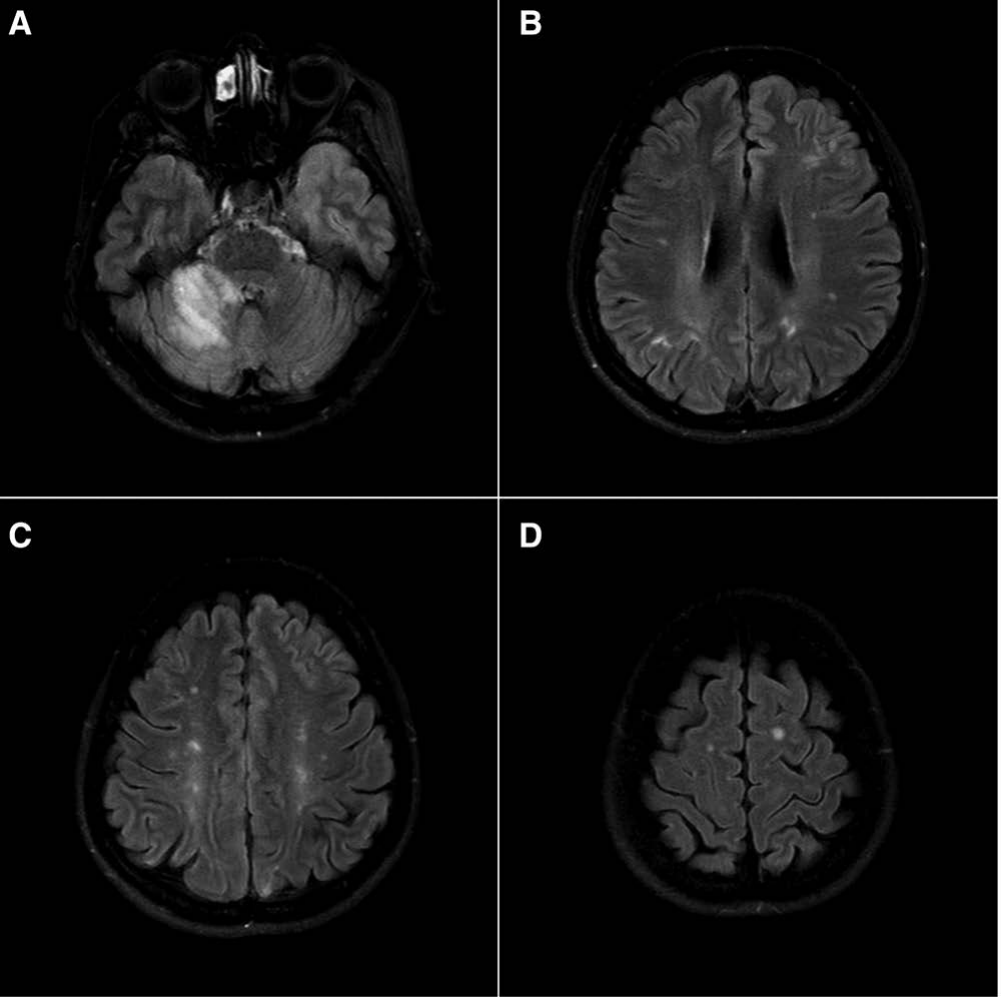
Brain magnetic resonance imaging. Multiple embolisms are observed in the cerebellum, larynx, and frontal and parietal lobes.

**Figure 2.**
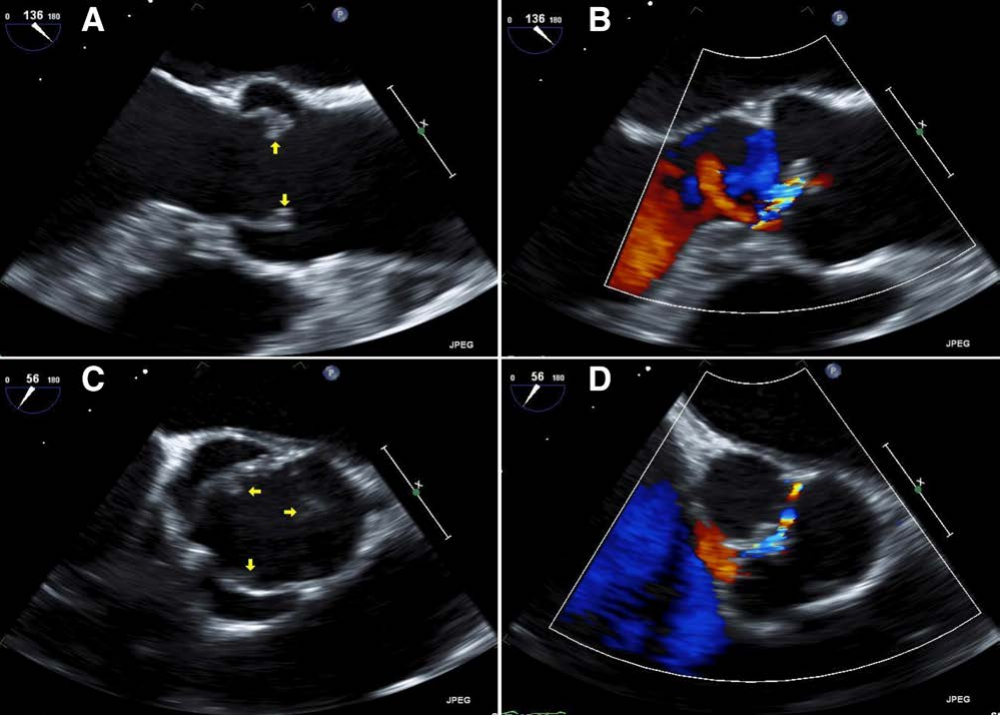
Transthoracic and transesophageal echocardiography. A mass adhering to the 3 leaflets is observed at the junction of the aortic valve.

Brain magnetic resonance imaging revealed multiple embolisms in the right cerebellum and the bilateral frontal, occipital, and parietal lobes (Fig. 1). Atherosclerosis was not observed on magnetic resonance angiography and carotid ultrasound. Significant arrhythmia was not observed on 24-hour ambulatory electrocardiographic monitoring. Transthoracic and transesophageal echocardiography were performed to evaluate for cardiogenic embolic stroke. The left ventricular ejection fraction was 58%. There were no thrombi in the left ventricle and atrium or the left atrial appendage. Agitated saline contrast used during transesophageal echocardiography did not reveal definite intracardiac shunts such as patent foramen ovale or pulmonary arteriovenous malformation. Transthoracic and transesophageal echocardiography revealed multiple echogenic masses attached the aortic leaflet, measuring 0.346 cm on the non-coronary cusp, 0.334 cm on the right coronary cusp, and 0.431 cm on the left coronary cusp (Fig. 2). Color Doppler echocardiography showed mild aortic regurgitation. The blood culture was negative for bacterial growth, and there was no serologic evidence of infection based on a C-reactive protein level of 0.26 mg/dL (reference, ≤0.3 mg/dL) and an erythrocyte sedimentation rate of 19 mm/h (reference, 0–30 mm/h). The definitive diagnosis was embolic cerebral infarction associated with NBTE.

**Figure 3.**
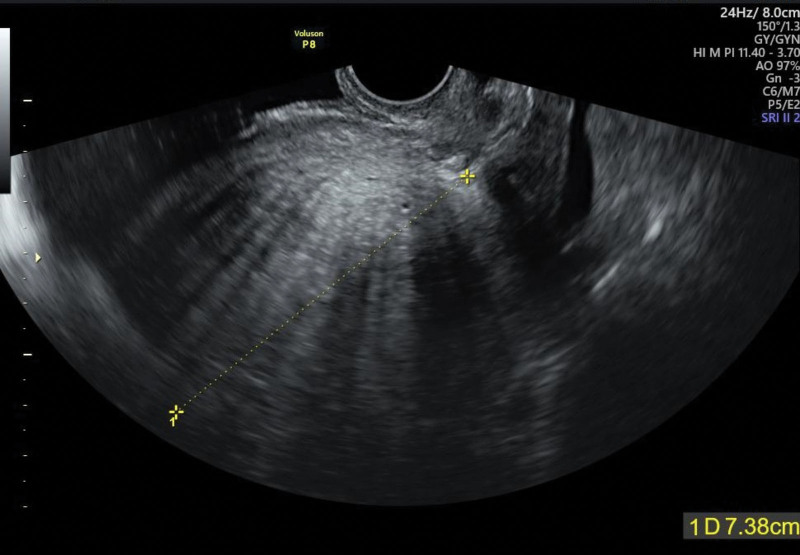
Uterine ultrasound. Note the thickening of the uterine wall and the relatively uniform echogenicity.

Tumor markers, coagulation factors, and autoimmune antibodies were evaluated to identify the cause of NBTE. The levels of cancer antigen 125 (CA125) and carbohydrate antigen 19-9 (CA19-9) were slightly elevated at 48.0 U/mL (reference, ≤35 U/mL) and 49.2 U/mL (reference, ≤34 U/mL), respectively (Table 1). The levels of fibrinogen degradation products and D-dimer levels were 30 µg/mL (reference, ≤5.0 µg/mL) and 13.66 µg/mL FEU (reference ≤ 0.55 µg/mL FEU), respectively; however, the levels of other coagulopathy-related blood parameters and autoimmune antibodies were within the normal range. Uterine ultrasonography performed to elucidate the cause of menorrhagia and the elevated CA125 and CA19-9 levels revealed a globally hypertrophied and homogenous uterus, suggesting adenomyosis (Fig. 3). Due to the lack of evidence for malignant tumors or hematologic diseases, adenomyosis was determined as the cause of NBTE.

**Table 1:** Laboratory results.

	Result	Reference
Cancer antigen 125 (U/mL)	48	≤35
Carbohydrate antigen 19-9 (U/mL)	49.2	≤34
Fibrinogen degradation production (µg/mL)	30	≤5.0
D-dimer (µg/mL FEU)	13.66	≤0.55
Fibrinogen (mg/dL)	315	170–380
Antithrombin III (%)	110.2	80–120
Cold agglutinin	Negative	≤1–16
Protein S activity (%)	61	55–123
Protein C activity (%)	91.3	70–140
Protein C antigen (%)	108	72–160
Protein S antigen (%)	96.7	60–150
Lipoprotein(a) (mg/dL)	16.9	≤31
C3 (mg/dL)	108.3	90–180
C4 (mg/dL)	25.5	10–40
Lupus anticoagulant antibody	Negative	
IgG antiphospholipid antibody (U/mL)	1.0	<10
IgM antiphospholipid antibody (U/mL)	2.0	<10
Antinuclear antibody	Negative	
IgG anticardiolipin antibody (U/mL)	0	
IgM anticardiolipin antibody (U/mL)	3.45	
Antineutrophil cytoplasmic antibodies	Negative	
Homocysteine (µmol/L)	8.2	<14.0

Medroxyprogesterone 20 mg/d and warfarin (target INR 2.0–3.0) were administered to treat adenomyosis and NBTE. The follow-up transesophageal ultrasound 1 month after treatment initiation indicated a slight reduction in the size of the vegetations, which did not completely disappear. Furthermore, despite hormone therapy, during the next menstrual period, menorrhagia worsened due to anticoagulation and transfusion of red blood cell was repeatedly required. A total of 5 pints red blood cell transfusions were performed. She underwent total laparoscopic hysterectomy. Although vegetations did not completely disappear even after surgery, no new infarctions were noted in the absence of anticoagulant treatment during the 3-year follow-up period.

## 3. Discussion and conclusion

In this case report, we presented a middle-aged woman with adenomyosis who developed cerebral and cerebellar embolic infarctions associated with NBTE. The patient did not experience recurrent cerebral infarction after hysterectomy, raising the possibility that adenomyosis as the cause for NBTE and embolic infarction.

Adenomyosis, which typically presents with menorrhagia and dysmenorrhea, is common in middle-aged women.^[1]^ However, our literature review identified a total of 19 reported cases of cerebral infarction related to adenomyosis, including the present case (Table 2).^[2–13]^ Although the definite mechanism underlying adenomyosis-associated cerebral infarction is unclear, tissue factor, which plays a role in coagulation and is associated with menorrhagia and dysmenorrhea, is elevated in the endometrium of patients with adenomyosis compared to healthy women. Another potential mechanism involves carcinoma mucins, including CA125 and CA19-9, that promote thrombosis through signaling in neutrophils and platelets. The evidence from the 19 cases reported to date provides support to this mechanism, given that cerebral infarction occurred during menstruation in 10 of the 18 patients; information on the relationship between the menstrual cycle and cerebral infarction was not available in the remaining 1 case. The levels of CA19-9 and CA125 were increased in all cases with available data, although the magnitude of increase differed across the cases.

**Table 2:** Review of cases of cerebral infarction associated adenomyosis.

Case	Author, year [reference]	Age	Region	Symptom	Recurrence	Onset during menstruation	CA125	CA19-9	D-dimer	Hb	NBTE	Location of vegetation	Treatment for cerebral infarction	Treatment for adenomyosis	Hysterectomy
1	Soeda S, 2011^[2]^	50	Japan	Hemianopsia	Yes	Yes	1519	NA	95.7	8.3	Yes	AV	No anticoagulation	GnRH agonist	Yes
2	Yamashiro K, 2012^[3]^	45	Japan	Hemiparesis, impaired consciousness	No	No	159	NA	1.1	8.4	No		UFH→antiplatelet	GnRH agonist	No
3	Yamashiro K, 2012^[3]^	44	Japan	Hemiplegia	No	No	NA	NA	NA	7	No		UFH→warfarin	GnRH agonist	No
4	Yamashiro K, 2012^[3]^	50	Japan	Hand weakness	No	Yes	42.6	NA	0.57	6.9	No		Aspirin	GnRH agonist	No
5	Yamashiro K, 2012^[3]^	42	Japan	Aphasia	Yes	Yes	1750	NA	6	8.6	No		1^st^-Antiplatelet 2^nd^-UFH→warfarin	GnRH agonist	No
6	Hijikata N, 2016^[4]^	59	Japan	Hand weakness	No	NA	334.8	NA	7.0	NA	Yes	AV	UFH	NA	NA
9	Aso Y, 2018^[5]^	44	Japan	Hand weakness, gait disturbance	Yes	Yes	2115	1824	17	10.3	No		1^st^-UFH→rivaroxaban 2^nd^-warfarin	GnRH agonist	Yes
8	Kim B, 2018^[6]^	49	Korea	Dysarthria, sensory change, hand weakness	No	No	379	69.2	3.99	9.9	Yes	MV	LMWH→warfarin		Yes
10	Okazaki K, 2018^[7]^	42	Japan	Hemiparesis, aphasia	No	Unknown	395	NA	1.4	NA	No		Warfarin	NA	
11	Okazaki K, 2018^[7]^	50	Japan	Hemiparesis, aphasia	No	Unknown	143	NA	3.7	NA	No		Rivaroxaban	NA	
7	Uchino K, 2018^[8]^	48	Japan	Hemiplegia, aphasia	No	No	901	1791	1.9	8.5	Yes	MV	UFH→warfarin		Yes
12	Yin X, 2018^[9]^	34	China	Vertigo	No	Yes	937.1	462.5	1.05	13.4	No		NA	NA	
13	Yin X, 2018^[9]^	37	China	Limb weakness	No	Yes	735.7	43.2	2.34	10.8	No		NA	NA	
14	Yin X, 2018^[9]^	46	China	Hemiplegia	No	Yes	546.5	1076.6	12.0	12.1	No		NA		Yes
15	Zhao Y, 2020^[10]^	34	China	Fever, limb weakness	No	Yes	937.7	Normal range	27.4	11.2	No		LMWH→clopidogrel	NA	No
16	Aiura R, 2021^[11]^	48	Japan	Impaired consciousness	No	Yes	3536.2	892.1	79.3	8.2	No		UFH→edoxaban		Yes
18	Arai N, 2022^[12]^	50	Japan	Hemianopsia	Yes	Yes	999	112	6.4	9.2	No		UFH→apixaban	GnRH agonist	Yes
17	Yasuda M, 2022^[13]^	47	Japan	Hand weakness, aphasia	Yes	Yes	90.3	52.3	3.8	11.3	No		UFH→edoxaban		Yes
19	19	47	Korea	Vertigo, impaired consciousness	No	Yes	48.0	49.2	13.66	3.4	Yes	AV	Warfarin	Medroxyprogesterone	Yes

AV = aortic valve, GnRH = gonadotropin-releasing hormone, Hb = hemoglobin, LMWH = low molecular weight heparin, MV = mitral valve, NA = not available, NBTE = nonbacterial thrombotic endocarditis, UFH = unfractionated heparin.

NBTE is considered as a possible mechanism of cerebral infarction in patients with adenomyosis. NBTE was present in 5 of the 19 reported patients with adenomyosis who were diagnosed with cerebral infarction.^[2,4,6,8]^ In NBTE, a mass with aseptic proliferation is attached to a cardiac valve. The prevalence of NBTE is 0.3% to 9.3%, and the incidence of embolism in patients with NBTE varies from 14.1% to 90.9%. The most common risk is advanced malignancy, especially adenocarcinoma of the lung or the ovary, followed by systemic lupus erythematous; it rarely occurs in tuberculosis, uremia, human immunodeficiency virus infection, antiphospholipid syndrome, rheumatoid arthritis, sepsis, and burn. Although the NBTE pathogenesis is not clear, endothelial cells have been demonstrated to be damaged by circulating cytokines such as tumor necrosis factor-alpha or interleukin 1, resulting in subendothelial connective tissue exposure to circulating platelets. Unlike infectious endocarditis, the vegetations in NBTE induce minimal inflammatory response, easily detach, and are therefore considered as more likely to systemically embolize; therefore, in patients with NBTE, embolic infarction is often the first clinical symptom. It is critical to treat the underlying cause and anticoagulant therapy is necessary in the absence of contraindications.

Currently, there are no guidelines on the type and duration of anticoagulant therapy for recurrence prevention in patients with adenomyosis-related cerebral infarction. The decision to initiate anticoagulant therapy is challenging even in patients with adenomyosis-related cerebral infarction and confirmed NBTE, such as the present case. First, recurrent cerebral infarction was reported in several similar cases despite the use of anticoagulants. Additionally, the use of anticoagulants can exacerbate menorrhagia, a typical symptom of adenomyosis. In the present case, hysterectomy was a definitive treatment approach to address adenomyosis and to prevent recurrence of cerebral infarction.

Interestingly, all 19 cases of adenomyosis with cerebral infarction were reported in East Asian countries, including 13, 4, and 2 cases in Japan, China, and Korea, respectively. Whether adenomyosis-associated cerebral infarction is related to racial or regional characteristics remains unclear, although some patients with adenomyosis harbor genetic polymorphisms, with several genes detected more frequently in Asian women. Polymorphisms in *COMT, MMP*, and *TIMP* have been reported as significant risk factors for adenomyosis in Asian populations.^[14–20]^ Further studies are needed to determine whether these are also risk factors for coagulopathy, NBTE, and systemic embolization and to assess whether other genetic factors are involved.

The present case adds to the limited number of previously reported cases and supports that, albeit rare, adenomyosis can be associated with embolic infarction and suggests that NBTE might be the link between adenomyosis and embolic infarction. This specific presentation has been reported only in Asian women, and future studies are warranted to elucidate the underlying genetic and environmental factors.

## Acknowledgments

The authors would like to thank Enago (www.enago.co.kr) for the English language review.

## Author contributions

**Conceptualization:** Jeong-Sook Seo.

**Data curation:** Jeong-Sook Seo.

**Formal analysis:** Jeong-Sook Seo.

**Investigation:** Jeong-Sook Seo.

**Methodology:** Jeong-Sook Seo.

**Project administration:** Jeong-Sook Seo.

**Resources:** Jeong-Sook Seo.

**Supervision:** Jeong-Sook Seo.

**Validation:** Jeong-Sook Seo.

**Visualization:** Jeong-Sook Seo.

**Writing – original draft:** Jeong-Sook Seo.

**Writing – review & editing:** Jeong-Sook Seo.
